# Guidelines and best practices for assessing young children remotely

**DOI:** 10.3389/fpubh.2024.1376090

**Published:** 2024-06-13

**Authors:** Kyla Z. McRoy, Lori E. Skibbe, Sammy F. Ahmed, Burcu H. Tatar

**Affiliations:** ^1^Early Language and Literacy Investigations Lab, Department of Human Development and Family Science, Michigan State University, East Lansing, MI, United States; ^2^Department of Human Development and Family Science, University of Rhode Island, Kingston, RI, United States

**Keywords:** preschool, assessment, remote, children, development

## Abstract

Given the recent rise in the use of remote assessments to collect data from young children, researchers and practitioners would benefit from guidance on best practices within the field. Based on our experiences with assessing over 600 preschoolers remotely, our research team provides a set of main principles to guide professionals to successfully create and operationalize systems for remote assessment. Guidelines include detailed information about how to choose a technology platform, select and use online assessments, and how to adapt traditional tasks for remote use. We also note the challenges inherent in using certain types of tasks, provide tips for scheduling remote sessions, and offer advice for how to promote children’s engagement throughout the assessment process.

## Introduction

Remote assessment is increasingly recognized as a valid and reliable method of conducting assessments with young children [e.g., (1)]. The utility of remote assessments has certainly been highlighted by the recent COVID-19 pandemic (2), and would be similarly valuable in other crises that cause school closures worldwide (e.g., earthquakes, viruses, teacher strikes, weather-related events). These crises result in large learning losses (3) that most strongly impact young children, dual-language-learners, and children of color (4). In these scenarios, evaluations (e.g., speech and language) cannot wait, as they are critical for early intervention (5). However, the benefits of remote assessments can persist even in non-crisis times.

Remote assessment can prevent delays in and increase access to diagnostic and screening services which allow children to receive treatments as needed [e.g., (6, 7)]. These methods can also allow flexibility for professionals working with young children to gather data to track educational progress and inform program development to better serve the needs of children [e.g., (8)]. Additionally, remote approaches can reduce the burden of in-person testing on families and educators, such as time and travel costs; this can further increase access to hard-to-reach and underserved populations (9, 10). Tele-assessment can also increase access to children with physical and health conditions that might exclude them from participating in traditional assessment approaches (11, 12). Finally, remote assessment capitalizes on technology that tends to be familiar and accessible to both children and families, sidestepping extensive training. Even young children can navigate apps and games on tablets successfully (13). Preschoolers have been observed to show high task completion and accuracy in field settings when using tablets (14). Educators have further reported their own and children’s enjoyment of remote assessment, as well as ease of use (9, 15).

Conducting remote assessments effectively with young children requires a different approach than that used with traditional paper-and-pencil assessments. Our research team moved to a remote testing platform in the fall of 2020, as many schools restricted visitors or began online instruction. Between then and the fall of 2021, we assessed over 600 preschoolers remotely in partnership with their schools. Based on our experiences, we provide a set of main principles to guide professionals who work with young children–including researchers, speech/language pathologists, early interventionists, behavioral consultants, and other practitioners–as they create effective systems for assessment. Although the current review aims to present guidance which can be adapted by researchers and practitioners to fit their needs, those seeking a detailed description of data collection, analysis, and evaluation of the psychometric properties of the specific measures used by our team can [see (1)], which examines a subset of the aforementioned data.

## Technological considerations

Certain technological features are required for remote assessment, such as reliable internet and two-way audio which allows an assessor to make contact with a caregiver to conduct the assessment. Similarly, webcams are necessary to allow children to see the assessor’s face and connect with them. Although some research teams recommend having two video screens (16), we were successful using only one. Researchers and practitioners should know that a remote platform may make it more challenging to safeguard their participants’ personal health information, which is protected by the Health Insurance Portability and Accountability Act (HIPAA). Personal health information includes information that is often collected by schools, such as the following: allergies, medications, immunization history, family history, and diagnoses. The software packages used for remote communication are often less secure, which can make it more challenging to keep any personal health information collected private (17). See Braden (18) for recommendations on HIPAA compliant software that can be used to conduct assessments if personal health information is being collected.

It is important to note that the use of remote assessment does not inherently provide equitable access for all families and children. Rather, remote assessment is a tool that–if used with thoughtful consideration–can promote the inclusion of underrepresented populations. Not all families have access to the technology required by some remote assessment methods (e.g., computer access, secure WiFi), so researchers must consider whether entities can help to provide equipment and internet access when needed, including partnerships with local libraries and participants’ schools/programs which may provide free technological access or equipment (e.g., tablets, hotspots) for rent at low cost. Researchers working with rural and remote schools have reported success with holding sessions at the school where teachers guide children to connect with remote assessors via school technology (9).

## Selecting assessments

It is important to ensure that tasks are developmentally appropriate and engaging for young children. Researchers and practitioners should confirm that any tasks chosen work in the ways intended, with evidence of good validity and reliability. They should also ensure that measures have been used across different racial and ethnic groups. If working with dual language or non-English speaking children, it is best if measures are available in the student’s preferred language(s) if possible.

### Using existing on-line assessments

Some assessments have already been adapted for online administration (see Table 1 for examples). Typically, these types of measures are housed on website platforms that automatically collect participant data as the task progresses, reducing the demand on the assessor to conduct proper scoring or store hard copies of data. Similarly, these tasks have built-in stopping rules, practice items, and other task-specific features that an assessor does not need to learn or manage. Some on-line assessments (e.g., ATLAS^1^) are even adaptive, meaning that each individual answers a set of items that are tailored to them, thus reducing testing time. We found that these types of assessments work best when the child can complete them independently or with the help of a person in the same physical location. Although it is possible to use on-line assessments when the assessor and child are in separate locations, this proved to be challenging in practice. Children often needed some behavioral support (e.g., reminder to wait for the audio prompt before selecting a response), and the children we worked with often had different internet speeds than our assessors, which affected the timing of audio prompts.

**Table 1:** Sample assessments of school readiness for young children that are available on-line.

Assessments	Domain(s) assessed	Length	Age	Where to learn more
ATLAS	Phonological awareness	10 min	3–7 year olds, including children with speech language impairment	www.accesstoliteracy.com
IGDIs	Early literacy, early numeracy, and social–emotional skills	10 min for each domain	3–6 year olds	https://igdi.ku.edu/virtual-igdis/
Get Ready to Read!	Early literacy in English and Spanish	10–15 min	4 year olds	https://www.getreadytoread.org/
QUILS	Language competence	15–20 min	3–5 year-olds	www.quilscreener.com
TX-KEA	Executive function, language, literacy, STEM, socioemotional, academic motor skills	1–5 min per subtest or 24–29 min total	4–6 year-olds	https://public.cliengage.org/tools/assessment/tx-kea/

### Adapting traditional tasks

Ensuring reliability and validity is critical to successfully adapting a traditional task into a remote version. Studies that have compared tasks administered in-person vs. remotely tend to consistently document no significant differences in children’s data, as well as high reliability (intra- and inter-rater, test–retest, and internal consistency via mean interitem association). This includes studies examining child language samples during play (19), general growth and development (20), social–emotional skills (21), motor skills (22), and established language and literacy assessments (23, 24) including those administered with bilingual children (25). Similarly, a systematic review of 23 studies showed strong agreement between remote and in-person assessment for cognitive functions between 18 months and 18 years of age (26). Typically, inconsistencies seem to be exclusive to timed measures (24), as previously mentioned in our experience as well. As such, although there may be some exceptions, there is strong evidence indicating that many tasks across domains can be appropriately adapted to virtual formats.

Many traditional tasks require children to respond by touching visual stimuli, so we recommend using a platform that allows for the use of remote control. Zoom allows assessors to first connect with children on their webcam, then to share their screens so children can see any visual stimuli required by the task. With remote control, when the child touches their own tablet screen, the cursor moves to that location. This approach has been used successfully by teams who work with very young children (27). Having remote control access mimics the in-person experience of having a child point to a picture on the page, a requirement of many assessments developed for young children. For example, the Peabody Picture Vocabulary Test-5 (28) asks children to point to a picture that represents a stated word. Although some teams have noted that this approach can limit the quality and resolution of visual stimuli (29), simple visuals can be used without issue.

Some assessment materials distributed for remote use are in PDF format [e.g., Woodcock Johnson Tests of Achievement; (30)]. When using the remote control function in Zoom, children’s touches can cause the document to scroll off the target item, skip items, revisit old items, zoom in/out, and/or highlight text or images. Children can also access the toolbar accidentally, or even close or minimize the document window. We recommend that PDF stimuli be converted into PowerPoint, which can be done without jeopardizing the validity and reliability of the task (1). Specific guidelines are provided below.

Steps to Creating PowerPoint slides from PDFs

1. Scan visual stimuli from original assessment materials. Images can be “snipped” with a screenshot tool and copy/pasted into a PowerPoint slide.
2. Once on the slide, images can be cleaned up with PowerPoint’s image tools if quality was lost during the scan.
3. Present one item per slide, as stacking items can be distracting to children.
4. To ensure that assessors can track the progression of task items, the corner of every slide can be marked with a small, borderless textbox that indicates the item number in subtle gray text.
5. Between slides, a brief fade-to-black transition can be used to help children understand when a new item has been presented.

Once the assessor shares their screen with the child, PowerPoint presentations can be made full-screen and the assessor can give the child remote control. Most slides will advance on a click, which can be done with a space bar, mouse, or a touch to the screen. If using remote control access, the PowerPoint file can be set to a spacebar or keyboard advance only, which will prevent children’s touches or clicks from advancing items outside of assessors’ control. Stimuli are generally presented full-screen, which prohibits the use of electronic scoring sheets (if only one screen is available). Thus, we asked our assessors to print out scoring sheets for use during the session.

Tasks that require gross motor movements can still be used, but assessors need to consider how children’s images are captured on the screen. For example, the Early Screening Inventory (31) includes tasks evaluating motor development where the child might move out of the frame (e.g., walking, hopping). In our experience with these kinds of tasks, children are not always able to position their tablets independently, but caregivers can hold up the tablet or place it against the wall at an angle to capture the child from head to toe. For tasks requiring the child to perform motor movements while remaining stationary, caregivers may place tape or sticky notes on the floor to give children a visual target so they remain in the frame.

## Types of assessments that are challenging to adapt

Some types of tasks proved difficult for us to use in the field. We found application-based tasks that used timed assessments to be incompatible with remote assessment. Since assessors and families had varying internet speeds across locations, we often experienced frequent lags and inaccuracies of recorded times. We also encountered difficulties scoring verbal responses from preschoolers accurately in the field. The intelligibility of young children’s speech is highly variable, although it is estimated that 55% of 3-year-olds speech is intelligible and 70% of 4-year-olds speech is intelligible (32). Although the audio quality of web platforms is usually strong, speech is not as clear in this setting as it is in person. For example, our team planned to ask children to say letter sounds (33). When piloting this in the field, our team found it difficult to hear differences between certain phonemes (e.g., /p/ and /b/). For other assessments, responses at the word level were not consistently intelligible (e.g., What rhymes with light?). These challenges were exacerbated when children wore masks during their assessment sessions, which muffled children’s voices. Remote testing children in their home environment sidesteps that issue, but we still advise caution when using remote tasks that require clear verbal responses from children.

## Scheduling remote assessments

Based on our experience, it can be difficult to effectively contact and schedule assessments with families. We found email communications to be unproductive and phone calls were often unsuccessful as well, so we sent texts and left voice messages. Families were most responsive to contact between 6 and 8 pm on weeknights or on Saturday mornings. Practitioners may choose to schedule assessments as part of a “virtual home visit” or remote parent-teacher conference to fold assessments into a familiar and standard practice, which may improve responsiveness. After a session was scheduled, we sent out several reminders to families via text message. This included an immediate reminder when the session was scheduled, a reminder the day before the testing session, and a final reminder 1 h before the session was supposed to begin. Nevertheless, the ‘no show’ rate for remote assessments conducted within the home was high. Researchers and practitioners may have to work hard to establish connections with families beforehand to help alleviate these issues.

## Conducting the assessment session

Having a caregiver present with a child during task administration helped engage families in the assessment process and allowed them to ask questions about their children’s development. Caregivers helped with camera placement if needed, directed children’s attention back to the screen, and repeated verbal responses if they were unclear to our assessors. This approach was particularly valuable when children needed behavioral supports, as is the case for many children with disabilities (BLINDED).

Assessors should be aware that parents often want to praise, help, or scold children, which can change the amount and types of information elicited from a child in ways that hinder valid assessment (34). The most common issue was reminding parents that children needed to complete tasks based on their own knowledge (35). Caregivers were reminded at the beginning of the session that performance-related feedback is not permitted, and that parents should ask children what they thought if they looked to them for help. Assessors provided reminders directed at parents (e.g., “We want to know what [child] knows all on her own!”) and children (e.g., “I want to know what *you* know!”). Assessors can give especially eager parents specific, appropriate phrases to encourage their children without threatening task validity; these are restricted to statements that praise effort (not performance), such as “You’re working so hard!” We also trained our assessors to enthusiastically praise children’s effort, as have other researchers (34).

Keeping a child engaged might require different techniques in a remote environment. In studies of remote learning, both teachers (36, 37) and parents (38) have reported challenges in maintaining children’s engagement and promoting participation online. Important aspects of in-person assessment that we find even more critical for remote assessment include using eye contact, speaking the child’s name, being playful and authentic, and breaking sessions into smaller pieces as needed. We have also gathered specific strategies that we believe to be effective in helping children to focus on remote tasks (note that it is always best to match strategies to children’s particular personalities and needs; see Table 2). Studies on remote learning in preschoolers have indicated that playful interactions like those in Table 1 can promote engagement even when online sessions run up to 30 min (39).

**Table 2:** Strategies for promoting focus and engagement during remote assessment.

Strategy	Description
Conversation	Particularly at the start of a session, we found it was critical to chat with the child and bond a little. Assessors might comment curiously about something the child is wearing (“I notice your shirt is red, is that your favorite color?”), ask what they were just doing in the classroom, or give a personal detail that sparks a conversation (“I just love dogs, do you have a dog at home?”).
“Look at me and make a silly face!”	An important element of remote testing–for interactions with both the assessor and tasks with visual stimuli–is keeping children’s eyes on the screen. Assessors may prompt a distracted child with this kind of engaging statement, which serves to both playfully re-engage the child and focus their gaze on the assessor or stimuli. This can be adapted to different statements (“look at me and stick out your tongue,” “look at me and make a fish face!”).
Toy Characters or Pets	Assessors can also make use of objects in their immediate environment to engage children. Several of our assessors used stuffed animals to create fun and supportive characters that the assessor could show on the screen as needed. If well-behaved pets are available to the assessor, they might perform a trick on screen between each task to motivate persistence; often just the appearance of a pet is motivating to children (e.g., “We finished that game, now we can see Jahi again!”).
“Where is my _______?”	One of our assessors put his glasses in a different location (e.g., head or shirt) between each task and then pretended to have lost them. Children found this quite funny and assisted him in locating them again. Aside from promoting engagement, this is a great way to build a relationship with children at the start of assessment. This strategy could also be used with a pen/pencil (in shirt pocket, behind ear, in hair, etc.).
Watching my Breath	Many practitioners may own (or have access to) an expandable breathing ball, which is a colorful plastic toy that can be pulled into a larger sphere or compressed back down. Shown by the assessor via Zoom, this may help a distracted child focus and breathe along with an assessor’s guidance. Other virtual tools can be used for this, such as a graphic or video for breathing (e.g., a balloon expanding and contracting). These can be found on YouTube by searching terms such as “breathing” and “mindfulness,” then adding “for kids.”
Encouraging Seated Movement	These include brief seated stretches or shake-outs, also used by other researchers in remote assessment sessions (34). We used these commonly with children, especially if we were concerned that a more dramatic physical reset (e.g., “dance break” below) might derail the session.
“Dance break!”	If a session is running long or a child is especially restless, the assessor could call a brief dance break and join the child in the activity (on screen). The assessor might model specific dance movements for the child to copy, or prompt the child to come up with movements that the assessor copies. If music is used, we recommend a brief song that has a clear “end” which prompts the child to sit and focus again. If music is not used, the assessor may need to end the dance break with a playful instruction to sit back down (e.g., “...and then we plop back down!”).
My Favorite Song	The assessor can ask about a preferred song, find that song online, and use the song to engage the child between tasks. For example, the assessor might finish the first task and then play the beginning of the song, encouraging the child to sing along; the assessor may sing along if they know the song, or ask the child to teach them the lyrics. Then the song is paused and the child must complete the second task, after which the song is resumed.
Stickers/Pictures	Stickers are often provided to children for completing assessments in-person, but virtual options also exist for this motivational tool. These can be downloaded from online sources, or the assessor may create their own ahead of time; this can be as simple as copy/pasting images into a Word document. Depending on time limitations and personal knowledge of the child, the assessor may pre-select (or choose live) images relevant to the child’s specific interests. To replicate the feel of choosing a sticker from a sheet, the assessor may provide a bank of options. The final collection can be shown to the child on screen at the end of assessment, or e-mailed to the caregiver. Some researchers have created their own graphics where a picture is colored in or revealed at different stages of completion (25).

Although all the above strategies may remain effective across diverse populations of young children, special attention may be needed to support dual language learners (DLLs) in remote work (25). DLLs are a key population because their learning is disproportionately affected by disruptions in schooling and they have reduced enrollment and attendance when learning options are remote (4, 40). We have assembled a few additional tips for remote work with DLLs, particularly if the assessment is not in their home language:

- Learn how caregivers prefer to communicate and use that platform (e.g., texting apps like WhatsApp or WeChat).
- Provide training videos and/or step-by-step instruction handouts with visuals to show how to use any required technology (tablets, etc.).
- Plan to allow more time for each assessment than may be required for a monolingual child; this may include breaking assessment batteries into even shorter sessions if possible to reduce fatigue.
- Whenever possible, select an assessor who is fluent (or at least familiar) with the child’s home language. At minimum, we recommend the assessor learns some words that might be relevant to testing (e.g., yes/no, thank you, or good job).
- If multiple sessions are needed, assign the same assessor to all the sessions. This can allow an assessor to build stronger relationships with children as well as caregivers, and communicate more effectively between sessions about necessary supports or adjustments.
- Ensure that assessors learn how to pronounce the child’s name correctly. This is both respectful and important for engagement, as correct pronunciation of a child’s name may facilitate connection and attention.

It is also critical to engage in broader culturally-responsive practices during remote sessions, regardless of language status [see (41) for details].

## Overall challenges of remote assessment

As mentioned, in our experience we identified several challenges of remote assessment including technology-related issues, scheduling and communication barriers, limitations on types of tasks amenable to adaptation, preventing parent influence, and promoting accessibility. Researchers and practitioners considering remote assessment should be aware of several additional overarching limitations of this methodology. Despite the promising findings of many studies, more formal validation studies are needed to provide stronger evidence for the psychometric soundness of virtual assessment, particularly for young children (42). Concerns have also been raised about the ability of remote assessment to capture nuance or observational richness, if needed for a particular measure. In addition, some studies show that parents prefer in-person assessment [e.g., (23)], which may hinder tele-assessment use in home environments.

## Conclusion

Recent research has highlighted the value of remote assessment with children and begun to offer guidance for its successful implementation (35). Most assessments that are commonly used in clinics and schools are amenable for use remotely. However, researchers and practitioners should keep in mind that remote assessments–while valuable–do not replace all other forms of screening or diagnostic assessments; it is important to gather information from multiple sources to inform diagnostic decisions (5). Similarly, we recommend avoiding attempts to use or adapt measures that rely on overly complex instructions or stimuli.

Remote research methods are continually evolving in response to technological advances, online measure creation, and accessibility improvements. In particular, the field is challenged by limited technology access both within the U.S. and internationally. Although this digital divide is often mentioned in recent remote-focused literature (43, 44), solutions remain elusive. To utilize the full potential of remote assessment, this issue must be directly addressed. In the meantime, researchers and practitioners may use the guidelines presented to identify a platform, select and adapt assessments as needed, and keep children engaged throughout the remote testing process.

## Author contributions

KM: Writing – original draft, Writing – review & editing. LS: Writing – original draft, Writing – review & editing. SA: Writing – original draft, Writing – review & editing. BT: Writing – original draft, Writing – review & editing.

## Footnotes

^1^www.accesstoliteracy.com
